# An Integrated Approach to the Exposome: Rappaport and Lioy Respond

**DOI:** 10.1289/ehp.1104719R

**Published:** 2012-03-01

**Authors:** Stephen M. Rappaport, Paul J. Lioy

**Affiliations:** School of Public Health, University of California, Berkeley, California, E-mail: srappaport@berkeley.edu; Environmental and Occupational Health Sciences Institute, Robert Wood Johnson Medical School–UMDNJ and Rutgers University, Piscataway, New Jersey

We welcome the remarks of van Tongeren and Cherrie regarding our recent editorial (Lioy and Rappaport 2011) and see no particular differences in our positions. As originally conceived, the exposome concept promoted investigations of disease etiology, that is, finding unknown causes of disease (Wild 2005). This requires an untargeted study design so that important, but as yet unrecognized, exposures will not be missed (Rappaport and Smith 2010). Such untargeted designs lend themselves to omic characterization of biospecimens (of the top-down type), as has been demonstrated in recent metabolomic investigations (e.g., Wang et al. 2011). Many external measurements of exposure focus on specific chemicals or classes of agents, but van Tongeren and Cherrie offer examples of untargeted designs (e.g., mining records of household food purchases). In any case, as measurements of external phenomena become less targeted, they become more exposomic (of the bottom-up type). The real issue is to recognize the underlying reasons for estimating exposure levels. If measurements are intended to find unknown sources of disease, then they are consistent with the exposome concept. If they are intended for other purposes (e.g., dose response, risk assessment/management, source characterization), then they follow more traditional lines of exposure assessment/science. As we emphasized in our editorial (Lioy and Rappaport 2011), both approaches have merit, and a combination of the two offers particular advantages for both identifying and preventing hazardous exposures, and thereby mitigating diseases.
